# Clinical outcomes of composite restorations with or without a glass ionomer liner after selective caries removal to soft dentin: a 36-month randomized controlled trial

**DOI:** 10.1007/s00784-026-07209-8

**Published:** 2026-11-05

**Authors:** Cecilia Muñoz-Sandoval, Juliana J. Jardim, Karla Gambetta-Tessini, Rodrigo A. Giacaman, Marisa Maltz

**Affiliations:** 1 Cariology Unit, Department of Oral Rehabilitation, Faculty of Dentistry, University of Talca, Talca, Chile; 2 Department of Preventive and Social Dentistry, Faculty of Odontology, Federal University of Rio Grande do Sul, Porto Alegre, RS Brazil

**Keywords:** Dental caries, Liner, Survival analysis, Composite resin, Selective caries removal, Soft dentin, Clinical trial

## Abstract

**Objectives:**

Selective caries removal to soft dentin (SCR-SD) is currently recommended for the management of deep dentine caries lesions in permanent teeth. However, whether a pulp-protective liner is required after SCR-SD remains uncertain. This randomized controlled trial compared the clinical performance and pulp vitality outcomes of composite resin restorations using a universal adhesive with or without a glass ionomer cement (GIC) liner after SCR-SD in deep caries lesions of permanent posterior teeth.

**Methods:**

A randomized controlled clinical trial was conducted in 144 deep dentin caries lesions from 98 participants aged 9 to 55 years. After SCR-SD, lesions were randomly allocated to one of two restorative strategies: a universal adhesive applied in selective enamel etching mode (SE) and composite resin without a GIC liner, or a conventional GIC liner followed by the same universal adhesive applied in selective enamel etching mode and composite resin (GIC + SE). Restorations were followed for 30–36 months. The primary outcome was restoration survival, assessed using selected FDI criteria. The secondary outcome was pulp vitality preservation. Survival analyses were performed using Weibull regression models.

**Results:**

Of the 144 treated lesions, 117 were evaluated after 30–36 months. Restoration survival did not differ significantly between groups, with survival rates of 96.0% for SE and 92.4% for GIC + SE (*p* = 0.37). In contrast, pulp vitality preservation was significantly higher in the SE group than in the GIC + SE group, with success rates of 97.5% and 87.3%, respectively (*p* = 0.01). In the adjusted Weibull regression model, SE was associated with a lower risk of pulp vitality failure compared with GIC + SE. Restorative failure was also strongly associated with pulp vitality failure.

**Conclusions:**

The use of a GIC liner after SCR-SD did not improve restoration survival and was not associated with better pulp vitality preservation. Composite restorations using universal adhesive applied in selective enamel etching mode placed directly over the remaining dentin showed favorable restorative and biological outcomes after 30–36 months. These findings suggest that routine liner placement after SCR-SD may not be necessary.

**Clinical significance:**

In deep dentin caries lesions treated with SCR-SD, placing a glass ionomer liner before composite restoration using a universal adhesive did not improve clinical outcomes. Effective sealing is achieved using universal adhesive applied in selective enamel etching mode and nanofilled composite resin. These simple clinical procedures maintained the restoration integrity and pulp vitality.

**Registered on **https://www.clinicaltrials.gov**(NCT04250142)**.

## Introduction

There is a wide global consensus that the management of cavitated deep caries lesions must be approached under the framework of minimal intervention dentistry, using conservative therapeutic approaches [1–4]. Among the minimally invasive therapies, selective caries removal to soft dentin (SCR-SD) has been progressively recognized as the standard of care for the treatment of deep caries lesions [1, 2, 4, 5]. This technique consists of partially removing the superficial compromised carious dentin, maintaining a thick layer of soft demineralized decayed dentin over the pulp, and completely removing carious tissues from the cavity walls [6]. The main advantage of this technique is to avoid pulp exposure, which can compromise tooth survival [7]. However, controversy still exists on selecting the most appropriate dental material for providing pulp protection and restoration longevity.

Following SCR-SD, different restorative strategies have been proposed to protect the pulp and ensure restoration longevity. Traditionally, cavity liners such as calcium hydroxide (CH) or glass ionomer cement (GIC) have been placed on the pulpal floor to provide a protective barrier against potential irritants [8]. However, debate persists as to whether preservation of the remaining dentin, combined with effective cavity sealing, is sufficient to maintain pulpal health without the use of specific lining materials [3].

Several randomized clinical trials (RCTs) have evaluated whether liner placement after SCR-SD influences pulp vitality preservation, postoperative symptoms, and restoration performance in permanent teeth. Across studies with follow-up periods ranging from 1 to 5 years, pulp vitality preservation has consistently remained high, generally between 94% and 100%, with no significant differences between restorations placed with calcium hydroxide, GIC, hydraulic calcium silicate materials, or without a liner [9–15]. Similarly, postoperative sensitivity, restoration quality, and other biological outcomes have remained comparable regardless of liner placement.

Medium-term clinical studies have also demonstrated favorable restoration survival following SCR-SD. Restoration survival has consistently remained high over follow-up periods of approximately 3 to 5 years, while preservation of residual carious dentin beneath a well-sealed restoration has not been associated with compromised restoration longevity [16–18]. Taken together, the available evidence suggests that routine liner placement provides little additional clinical benefit for either pulp vitality preservation or restoration performance. However, randomized clinical trials specifically designed to evaluate whether cavity liners improve restoration survival following SCR-SD remain scarce.

Current professional recommendations are not entirely consistent. The European Society of Endodontology (ESE) position statement supports the placement of a cavity liner after selective caries removal in deep lesions [8]. Subsequent ESE publications, however, have highlighted the need for greater clarification and consensus regarding the management of deep carious lesions [19]. Likewise, the recent International Association of Pediatric Dentistry (IAPD) consensus recommendations support liner placement during indirect pulp treatment [20]. Therefore, the clinical relevance of routine cavity liner placement following SCR-SD remains uncertain.

Therefore, this randomized controlled trial aimed to compare the clinical outcomes of composite resin restorations placed with or without a conventional glass ionomer liner after SCR-SD in deep caries lesions of permanent posterior teeth. The primary outcome was restoration survival, and the secondary outcome was pulp vitality preservation over a 30–36-month follow-up period. The primary null hypothesis was that restoration survival would not differ between restorations placed with and without a conventional glass ionomer liner. The secondary null hypothesis was that pulp vitality preservation would not differ between the two treatment groups.

## Materials and methods

### Study design and ethical considerations

A randomized controlled trial was conducted, registered at ClinicalTrials.gov (NCT04250142), and approved by the Ethics Committee of the University of Talca (Folio 11/2018). The sample included adults (*n* = 74) and minors (*n* = 24). Written informed consent and assent were obtained as appropriate. The study was conducted in accordance with the Declaration of Helsinki.

Participants received the assigned treatment at the Dental Clinics of the Faculty of Dentistry, University of Talca, Chile, between 2018 and 2019. Follow-up evaluations were originally planned at 1, 12, 24, and 36 months after treatment. However, due to the COVID-19 pandemic, the follow-up schedule was adjusted, and the final evaluation was performed at 30–36 months.

### Sample size

An alpha error of 0.05 and a power of 0.80 (80%) were selected and the teeth with carious lesions were considered as the sample unit. Differences between the study groups were estimated to be around 17% [21], with an equally distributed number of teeth in both groups. A 20% drop-out rate was anticipated. This sample size calculation resulted in 144 teeth (lesions) with carious lesions. Thus, a minimum of 71 teeth were needed in each group to conduct survival regression models.

### Participants and recruitment

Participants presenting deep caries lesions were invited to participate among those attending the University clinic, and informed about the study implications. Participants were recruited during the years 2018 and 2019, and clinical examinations, treatments, and follow-up assessments were performed by a single clinician (CM).

**Inclusion criteria**:

1. Permanent molars or premolars with occlusal or proximal caries lesions radiographically classified as D2 or D3 were eligible [22]. D2 lesions involved 50–74% of the dentin thickness, whereas D3 lesions involved ≥ 75% of the dentin thickness toward the pulp.
2. Presence of pulp sensitivity assessed by thermal test using refrigerated gas (Endo Ice^®^ Refrigerant Spray, Coltene, USA).
3. Absence of history of spontaneous pain or pain at vertical and/or horizontal percussion.
4. Absence of a periapical lesion (assessed by periapical radiographs).
5. The carious lesion had to be primary, and the first choice of restorative treatment had to be direct composite resin (CR) restoration.

**Exclusion criteria**:

1. Uncontrolled systemic diseases detected during the anamnesis.
2. Cervical margins of the cavity involving dentin or root cementum.

### Randomization procedures

The randomization was computed using a software (http://www.random.org). The lesions listed were randomly assigned to 2 experimental groups once the SCR-SD was performed and the cavity was ready to receive the restoration. When participants presented more than one eligible lesion, each lesion was randomized independently. The study groups were divided according to the restorative treatment received: Group 1: SCR-SD + SE (universal adhesive applied in selective enamel etching mode, Single Bond Universal Adhesive, 3 M Oral Care, Saint Paul, MN, USA)^1^ + CR (Filtek Z350 XT^®^, 3 M Oral Care, USA)^2^, Group 2: SCR-SD + GIC (Ketac Molar, 3 M Oral Care, USA) + SE (universal adhesive applied in selective enamel etching mode, Single Bond Universal Adhesive, 3 M Oral Care, Saint Paul, MN, USA) + CR (Filtek Z350 XT^®^, 3 M Oral Care, USA).

### Intervention

Teeth were treated under absolute isolation with rubber dam and under local anesthesia. The necrotic and disorganized tissues were removed with manual excavators until reaching soft dentine at the bottom of the cavity and leaving the remaining dentin in relation with the pulpal wall, as described [5]. **Group 1**: After SCR-SD, selective etching (enamel only) was performed with 35% orthophosphoric acid for 15 s (Ultra-etch, Ultradent Products Inc., South Jordan, UT, USA). The enamel was rinsed for 30 s and dried for 15 s. When proximal surfaces were involved, a sectional matrix system (Composi-Tight^®^ 3D XR Sectional Matrix System, Garrison Dental Solutions, Spring Lake, MI, USA) was placed, with a proximal wedge, when needed. Single Bond Universal Adhesive (3 M Oral Care), a universal adhesive, was applied in selective enamel etching mode by actively rubbing the cavity for 20 s and then gently air-drying for 5 s to evaporate the solvent. The adhesive layer was light-cured for 20 s using a light-curing unit (Woodpecker, Guilin, China), with a manufacturer-reported irradiance of 1000–1200 mW/cm². The cavity was restored with Filtek Z350 XT^®^ nanofilled composite resin (3 M Oral Care, St. Paul, MN, USA) using an incremental technique, with each increment light-cured for 20 s. Finally, the occlusion was checked and adjusted. Final polishing was performed at the following appointment (one week later) using finishing fine-grind diamond burs (No. 1190 F, KG Sorensen, Cotia, SP, Brazil).

### Group 2

After SCR-SD, a conventional GIC liner (Ketac Molar™, 3 M Oral Care, St. Paul, MN, USA) was manually prepared according to the manufacturer’s instructions and applied to the cavity floor. Subsequently, enamel was selectively etched with 35% orthophosphoric acid for 15 s. When proximal surfaces were involved, a sectional matrix system (Garrison Dental Solutions, Spring Lake, MI, USA) was placed. The Single Bond Universal Adhesive and Filtek Z350 XT^®^ composite resin were then applied as previously described for Group 1.

Once the restoration was completed, a periapical and a standardized bitewing radiograph were taken for each restoration.

### Clinical assessment

A single trained clinician (CM) performed the final clinical evaluations at the 30–36-month follow-up. Selected FDI criteria for the direct clinical evaluation of restorations were used [23]. The assessed functional properties included fracture and retention, marginal adaptation, and radiographic findings. The assessed biological properties included pulp vitality, secondary caries, erosion, abfraction, tooth integrity, and periodontal response. Pulp vitality was evaluated based on patient-reported symptoms, response to thermal testing, absence of periapical lesions, absence of infectious processes such as sinus tract or fistula, and absence of pain on percussion. Each criterion was scored using the five FDI categories, ranging from clinically excellent to clinically poor.

### Radiographic evaluation

The radiographic examination was performed by an experienced radiologist, using periapical X-rays and bitewing radiographs taken at baseline and 30–36 months after restoration. Periapical lesions and proximal carious lesions occurrence were evaluated.

### Training and clinical calibration of the clinical examiner

For SCR-SD, the operator received a detailed explanation of the criteria and techniques used and was trained by an experienced clinician on the SCR-SD technique (JJ). For the FDI criteria, the examiner was trained with clinical photographs of the restorations. For the evaluation of restorations according to the FDI criteria [23], the clinical examiner (CM) underwent calibration using 25 standardized clinical photographs previously scored by a gold standard examiner (RAG). Inter- and intra-examiner agreement obtained during this calibration exercise were 0.73 and 0.77, respectively, indicating substantial agreement [24].

### Blinding

Participants were unaware of the restorative strategy used, and the statistician was blinded to participant identity and treatment allocation. Owing to logistical constraints during the extended follow-up period, the final clinical assessments were performed by the same trained clinician (CM) who had provided the treatment. To minimize observer bias, the examiner had no access to previous clinical records or treatment allocation and evaluated all restorations according to predefined FDI criteria.

### Data collection

Plaque accumulation was assessed using the O’Leary Plaque Control Record, and gingival inflammation was assessed as the percentage of sites with bleeding on probing (BOP). Factors potentially associated with restoration failure and pulp vitality failure were evaluated, including sociodemographic and clinical variables: sex (female or male), age (≤ 23 years or > 23 years), plaque index (< 85% or ≥ 85%), bleeding on probing (< 40% or ≥ 40%), decayed, missing and filled teeth index (DMFT; <6 or ≥ 6), affected surface (occlusal or occlusal-proximal), dentin lesion depth (50–74% or ≥ 75% of dentin thickness), arch (upper or lower), and tooth type (molar or premolar). Cut-off values for visible plaque index, gingival bleeding index, and DMFT were defined according to the median values observed in the study sample. This data-driven categorization was used to obtain balanced comparison groups for the exploratory analysis of factors potentially associated with clinical outcomes, rather than to represent established clinical thresholds for caries or periodontal risk.

### Data analysis

The primary outcome was restoration failure, defined according to the functional properties of the FDI criteria, including fracture and retention, marginal adaptation, and radiographic findings. The secondary outcome was pulp vitality failure, defined according to the biological properties of the FDI criteria, including loss of pulp sensitivity, symptoms compatible with irreversible pulpitis, periapical pathology, infectious processes, or pain on percussion. According to the FDI criteria, restorations are classified on a five-point scale ranging from 1 (clinically excellent) to 5 (clinically poor). In the present study, failures were recorded only when the corresponding FDI criterion was categorized as “poor” (score 5).

The distribution of baseline characteristics between study groups was compared using the chi-square test or Fisher’s exact test, as appropriate. Restoration survival and pulp vitality preservation were analyzed using Weibull regression models. Demographic and oral health variables were explored as potential predictors. Median values were used to dichotomize plaque index, gingival bleeding index, and DMFT. For DMFT, ICDAS 5 and 6 lesions were considered as the “D” component [25].

Weibull regression models were used to address the primary and secondary hypotheses by comparing time to restoration failure and pulp vitality failure, respectively, between treatment groups. Additional demographic, oral health, and lesion-related variables were explored as potential predictors of each outcome. Unadjusted models were first fitted for each variable, followed by adjusted models to estimate the association between treatment group and each outcome while accounting for selected covariates. Analyses were performed using STATA software, version 13.0. The significance level was set at 5%.

## Results

**Fig. 1:**
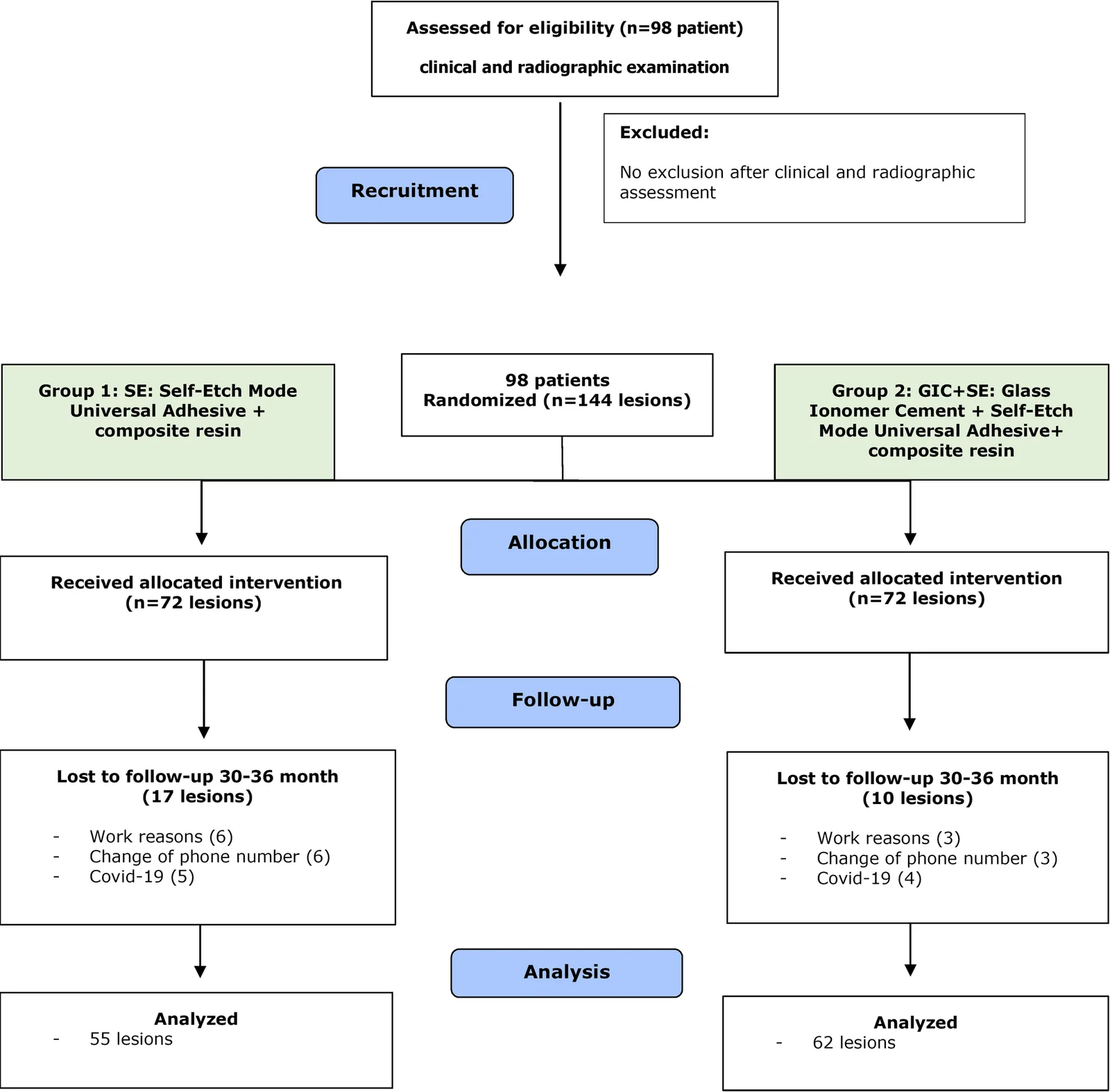
Flow diagram of the study

SCR-SD was performed in 144 deep caries lesions from 98 participants aged 9 to 55 years (median: 24 years). Most treated lesions were from female participants (52.1%, *n* = 75) and from participants aged ≤ 23 years (53.5%, *n* = 77). Of the 144 treated lesions, 117 were evaluated at the 30–36-month follow-up in 83 participants. Lesions lost to follow-up (*n* = 27; 18.8%) were mainly due to changes of address, work-related constraints, or refusal to attend the follow-up visit, particularly during the COVID-19 pandemic (Fig. 1). Among the 117 evaluated restorations, 47 (40.2%) were placed in premolars and 70 (59.8%) in molars. Thirty-three restorations (28.2%) involved occlusal surfaces, whereas 84 (71.8%) involved occlusal-proximal surfaces. Baseline sociodemographic and clinical characteristics according to treatment group are shown in Table 1.

**Table 1:** Baseline distribution of study variables by participants and included lesions according to treatment group

	Variable		Total n (%)	Cavity liner		*p* value
				SE n (%) 72 (50.0)	GIC + SE n (%) 72 (50.0)	
Participants ( *n*= 98)	Gender	Male	46 (46.9)	24 (52.2)	22 (47.8)	0.43
		Female	52 (53.1)	23 (44.2)	29 (55.8)	
	Age	≤ 23 years	49 (50.0)	24 (48.9)	25 (51.1)	0.84
		≥ 23 years	49 (50.0)	23 (46.9)	26 (53.1)	
	Plaque index	< 85	39 (39.8)	19 (48.7)	20 (51.3)	0.90
		≥ 85	59 (60.2)	28 (47.5)	21 (52.5)	
	Gingival bleeding index	< 40	50 (51.0)	23 (46.0)	27 (54.0)	0.69
		≥ 40	48 (49.0)	24 (50.0)	24 (50.0)	
	DMFT	< 6	49 (50.0)	26 (53.1)	23 (46.9)	0.31
		≥ 6	49 (50.0)	21 (42.9)	28 (57.1)	
Lesions (*n* = 144)	Restoration type	Premolar	63 (43.8)	46 (56.8)	35 (43.2)	0.07
		Molar	81 (56.2)	26 (41.3)	37 (58.7)	
	Surface type	Occlusal	37 (25.7)	22 (59.5)	15 (40.5)	0.18
		Occlusal-proximal	107 (74.3)	50 (46.7)	57 (53.3)	
	Dentine Lesion Depth	50–74%	82 (56.9)	36 (43.9)	46 (56.1)	0.09
		> 75%	62 (43.1)	36 (58.1)	26 (41.9)	
	Arch	Upper	88 (61.1)	39 (44.3)	49 (55.7)	0.09
		Lower	56 (38.9)	33 (58.9)	23 (41.1)	

SE: Selective Enamel Etching Mode Universal Adhesive, GIC + SE: Glass Ionomer Cement + Selective Enamel Etching Mode Universal Adhesive

Chi-square test

Most restorations remained clinically acceptable after 30–36 months of follow-up. Restoration failure was observed in 11 teeth (9.4%), whereas 106 restorations (90.6%) were classified as successful according to the functional FDI criteria. Four restoration failures occurred in the SE group and seven in the GIC + SE group. The reasons for restoration failure included material fracture, marginal adaptation defects, and radiographic findings. No restoration failures were attributed to secondary caries, erosion, abfraction, tooth integrity, or periodontal response.

Radiographic assessment was available for all 117 restorations evaluated at the 30–36-month follow-up. According to the selected FDI radiographic criterion, 109 restorations (93.2%) were classified as excellent and eight (6.8%) as poor. In the SE group, 53 of 55 restorations (96.4%) were classified as excellent and two (3.6%) as poor. In the GIC + SE group, 56 of 62 restorations (90.3%) were classified as excellent and six (9.7%) as poor. These radiographic classifications contributed to the primary restoration outcome and were not considered a separate outcome (data not shown).

**Fig. 2:**
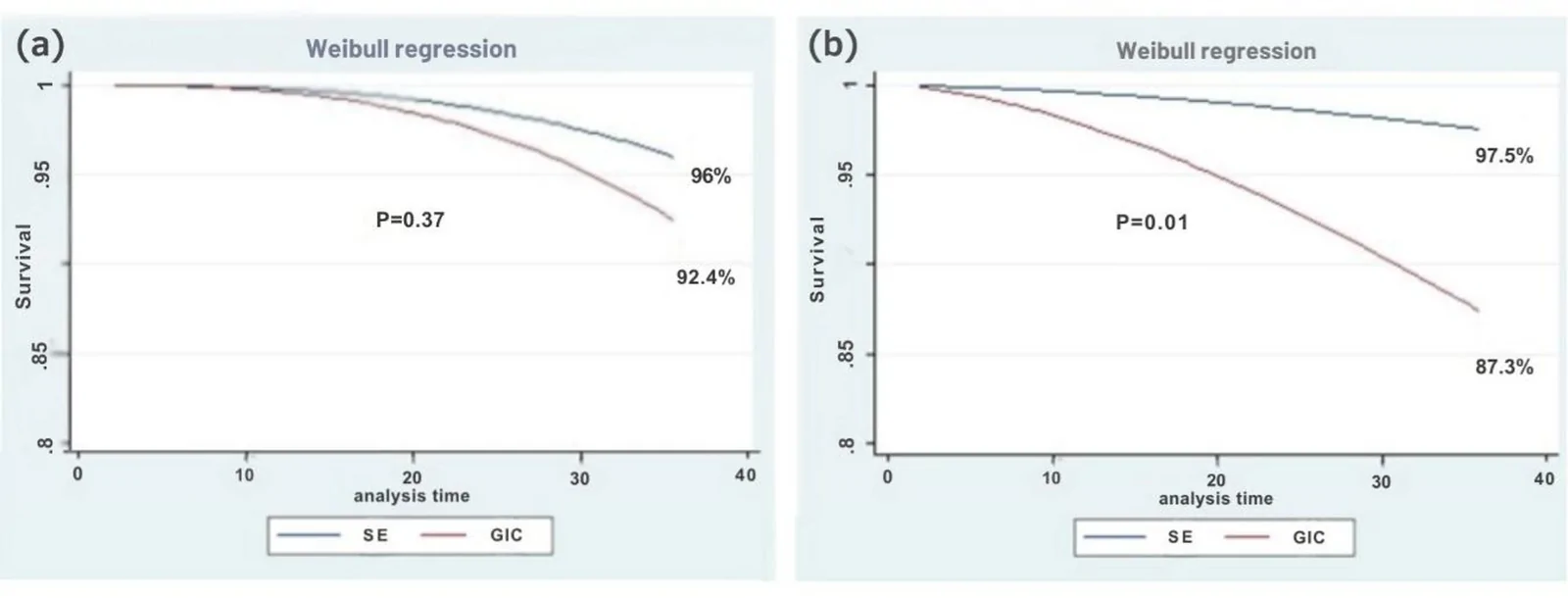
(**a**) Survival rates for composite resin restorations and (**b**) survival rates for pulp vitality after selective caries removal to soft dentin (SCR-SD), according to the use or non-use of a cavity liner (SE or GIC + SE), during the 30–36-month follow-up period (Weibull regression model)

Restoration survival did not differ significantly between groups. The estimated survival rate was 96.0% for restorations placed with SE and 92.4% for those placed with GIC + SE (*p* = 0.37; Fig. 2a). In both the unadjusted and adjusted Weibull regression models, no variable was significantly associated with restoration failure (Table 2).

**Table 2:** Association between restorative failures and explanatory variables (unadjusted and adjusted Weibull regression models)

Variables		Unadjusted		Adjusted	
		HR (95% CI)	*p*	HR (95% CI)	*p*
Gender	Male (*n* = 57)	1.00			
	Female (*n* = 60)	1.17 (0.35–3.87)	0.80		
Age	≤ 23 years (*n* = 63)	1.00			
	≥ 23 years (*n* = 64)	0.71 (0.21–2.41)	0.58		
Tooth type	Molar (*n* = 70)	1.00		1.00	
	Premolar (*n* = 47)	0.14 (0.02–1.15)	0.07	0.26 (0.02–2.87)	0.27
Surface type	Occlusal (*n* = 67)	1.00			
	Occlusal-proximal (*n* = 50)	1.80 (0.40–8.11)	0.44		
Plaque index	< 85 (*n* = 55)	1.00		1.00	
	≥ 85 (*n* = 62)	2.44 (0.66–9.01)	0.18	1.70 (0.43–6.68)	0.45
Gingival bleeding index	< 40 (*n* = 57)	1.00		1.00	
	≥ 40 (*n* = 60)	10.08 (1.29–78.95)	**0.02**	7.16 (0.95–53.82)	0.06
DFMT	< 6 (*n* = 68)	1.00		1.00	
	≥ 6 (*n* = 56)	1.37 (0.42–4.47)	0.60		
Liner	GIC + SE (*n* = 62)	1.00		1.00	
	SE (*n* = 55)	0.54 (0.16–1.79)	0.31	0.52 (0.12–2.17)	0.37
Dentine Lesion Depth	50–74% (*n* = 62)	1.00			
	> 75% (*n* = 55)	2.02 (0.59–6.86)	0.26		

*HR* = Hazard ratio; *CI* = Confidence interval

SE: Selective Enamel Etching ModeUniversal Adhesive, GIC + SE: Glass Ionomer Cement + Selective Enamel Etching Mode Universal Adhesive

Overall, most treated teeth remained vital after 30–36 months of follow-up (*n* = 103; 88.0%). Pulp vitality preservation was significantly higher in the SE group than in the GIC + SE group, with estimated success rates of 97.5% and 87.3%, respectively (*p* = 0.01; Fig. 2b). In the adjusted Weibull regression model, teeth restored with SE were less likely to experience pulp vitality failure (*n* = 2) than those restored with GIC + SE (*n* = 12) (HR = 0.18; 95% CI: 0.05–0.63; *p* = 0.01; Table 3). Of the 12 pulp vitality failures observed in the GIC + SE group, six were associated with restoration failure. Both pulp vitality failures observed in the SE group were also associated with restoration failure. Restoration failure was strongly associated with pulp vitality failure in the adjusted model (HR = 12.22; 95% CI: 3.82–39.17; *p* < 0.001; Table 3). Dentin lesion depth was not significantly associated with pulp vitality failure in either the unadjusted (*p* = 0.08) or adjusted model (*p* = 0.34).

**Table 3:** Association between pulpal failures and explanatory variables (unadjusted and adjusted Weibull regression models)

Variables		Unadjusted		Adjusted	
		HR (95% CI)	*p*	HR (95% CI)	*p*
Gender	Male (*n* = 57)	1.00			
	Female (*n* = 60)	0.96 (0.33–2.74)	0.94		
Age	≤ 23 years (*n* = 63)	1.00			
	≥ 23 years (*n* = 64)	0.92 (0.32–2.67)	0.88		
Tooth type	Molar (*n* = 70)	1.00			
	Premolar (*n* = 47)	0.82 (0.27–2.50)	0.73		
Surface type	Occlusal (*n* = 67)	1.00			
	Occlusal-proximal (*n* = 50)	2.42 (0.54–10.81)	0.25		
Plaque index	< 85 (*n* = 55)	1.00			
	≥ 85 (*n* = 62)	1.63 (0.55–4.89)	0.38		
Gingival bleeding index	< 40 (*n* = 57)	1.00		1.00	
	≥ 40 (*n* = 60)	3.65 (1.01–13.23)	**0.048**	2.08 (0.64–6.80)	0.22
DFMT	< 6 (*n* = 68)	1.00			
	≥ 6 (*n* = 56)	1.51 (0.52–4.36)	0.45		
Liner	GIC + SE (*n* = 62)	1.00		1.00	
	SE (*n* = 55)	0.16 (0.04–0.73)	**0.02**	0.18 (0.05–0.63)	**0.01**
Dentine Lesion Depth	50–74% (*n* = 62)	1.00		1.00	
	> 75% (*n* = 55)	2.87 (0.89–9.25)	0.08	1.66 (0.58–4.73)	0.34
Restorative failure	Absent (*n* = 106)	1.00		1.00	
	Present (*n* = 11)	18.12 (6.29–52.24)	**< 0.001**	12.22 (3.82–39.17)	**< 0.001**

*HR* = Hazard ratio; *CI* = Confidence interval

SE: Selective Enamel Etching Mode Universal Adhesive, GIC + SE: Glass Ionomer Cement + Selective Enamel Etching Mode Universal Adhesive

## Discussion

This RCT evaluated whether the use of a conventional GIC liner improves clinical outcomes after SCR-SD in deep caries lesions of posterior permanent teeth. The findings showed that GIC liner placement did not improve composite restoration survival compared to composite restorations using a universal adhesive applied in selective enamel etching mode placed without a GIC liner. In addition, teeth restored without a GIC liner showed significantly higher pulp vitality preservation over the 30–36-month follow-up. These results suggest that routine placement of a GIC liner after SCR-SD may not be necessary, provided that the cavity lesion is adequately sealed using a universal adhesive applied in selective enamel etching mode and nanofilled composite resin.

The difference in pulp vitality preservation should be interpreted in light of the strong association observed between restoration failure and pulp vitality failure. Although restoration survival did not differ significantly between groups, teeth with restoration failure had a markedly higher risk of pulp vitality failure. These finding reinforces the clinical relevance of maintaining restoration integrity after SCR-SD and suggests that pulpal outcomes may be closely linked to the quality and durability of the coronal seal.

The use of GIC or hydraulic calcium silicate as liners in deep caries lesions has been recommended by the European Society of Endodontology for pulp protection in cases without pulp exposure [8]. However, the available clinical evidence does not clearly support the superiority of any specific lining material. RCTs evaluating liner use after SCR-SD have consistently reported no significant benefit in terms of treatment success. For instance, Singh et al. found that the use of different liners did not influence clinical outcomes after 12 months, with comparable success rates even in restorations placed directly over carious dentin using a universal adhesive applied in self-etch mode [11]. Similarly, Azambuja et al. reported no differences in pulp vitality when indirect pulp protection was performed using either a calcium hydroxide liner or a universal adhesive, with consistent findings at extended follow-up [12]. In line with these results, studies assessing restorative outcomes have also shown that liner placement does not improve restoration performance after selective caries removal [13]. Differences in handling characteristics, polymerization mechanisms, and mechanical properties between conventional and resin-modified glass ionomer cements should also be considered when comparing clinical outcomes across studies [26].

Long-term evidence supports the concept that residual carious dentin beneath restorations does not compromise restoration longevity. Prospective and randomized studies have consistently shown comparable outcomes between selective and more invasive caries removal strategies. For example, Maltz et al. [27] reported restoration survival rates of 82% and 63% at 5 and 10 years, respectively, following SCR-SD in deep lesions. Similarly, Casagrande et al. found no differences in restoration longevity between complete and selective caries removal after 36 months [16], while Jardim et al. demonstrated that residual carious dentin did not negatively affect restoration survival over a 5-year period [17]. More recently, an RCT also showed that liner placement did not influence pulp vitality outcomes after SCR-SD at 5 years [14]. Taken together, these findings support the concept that preserving affected dentin and achieving an adequate coronal seal can provide favorable long-term clinical outcomes, without requiring routine liner placement after SCR-SD.

The present findings support the concept that effective sealing of the carious lesion is a more relevant determinant of pulp vitality than the placement of an intermediate lining material. This interpretation is consistent with higher-level evidence, as systematic reviews have reported limited or no benefit from the routine use of liners beneath composite resin restorations. A Cochrane review concluded that there is insufficient evidence to support the use of cavity liners for reducing postoperative sensitivity or improving restoration survival in Class I and II restorations [28]. Similarly, a systematic review and meta-analysis indicated that liners or bases do not enhance restoration longevity and may even negatively affect outcomes in certain clinical scenarios [29].

The favorable pulp vitality outcomes observed in the present study should also be interpreted in the context of the SCR-SD strategy itself. By preserving soft dentin adjacent to the pulp and avoiding unnecessary pulp exposure, SCR-SD helps maintain the integrity of the dentin–pulp complex, thereby preserving conditions that allow the tooth’s natural reparative response [3, 8]. Long-term clinical studies have also demonstrated that selectively preserving affected dentin beneath a well-sealed restoration does not compromise restoration longevity or pulp health [17, 18]. Therefore, the favorable biological outcomes observed in the present study are more likely related to the combined effect of a minimally invasive caries removal strategy and an effective coronal seal than to the biological properties of any restorative material alone.

Within this biological context, experimental studies have also explored the biological response of the pulp–dentin complex following the application of universal adhesives in self-etch mode. Unlike conventional self-etch adhesive systems, universal adhesives contain functional acidic monomers and solvents, allowing their use under different bonding strategies. Evidence from animal models suggests that the application of universal adhesives to deep dentin does not necessarily induce pulp necrosis or severe inflammatory responses and may support early pulp repair [30]. In addition, universal adhesives applied in self-etch mode have been associated with favorable biological responses [31, 32]. Growth factors such as transforming growth factor beta-1 (TGF-β1), which are involved in dentin repair and tertiary dentin formation, may be released or activated following different pulp-capping materials and restorative procedures. However, the biological role of these signaling pathways in pulp repair remains incompletely understood, and the available evidence is derived primarily from experimental models [33]. However, the biological role of these signaling pathways in pulp repair is not yet fully understood, and the available evidence is primarily derived from experimental models. In clinical conditions, factors such as dentin sclerosis and altered substrate characteristics may limit adhesive penetration and affect their biological performance [34].

Some limitations of this study should be acknowledged. The COVID-19 pandemic affected the planned follow-up schedule and contributed to participant attrition, which may have influenced outcome assessment. Although the initial sample size calculation accounted for the expected loss to follow-up and the observed attrition remained within this estimate. In addition, the relatively small number of restoration and pulp vitality failures may limit the precision of the regression estimates, particularly in the adjusted models. Because some participants contributed more than one lesion, potential clustering of lesions within participants should also be considered when interpreting the findings. Another limitation is that the treatment and outcome assessments were performed by the same clinician. Although the examiner was calibrated before the study, had no access to previous clinical records or treatment allocation during follow-up, and used predefined FDI criteria, the possibility of observer bias cannot be completely excluded. Finally, the absence of intermediate follow-up assessments limited the ability to determine the precise timing of restoration and pulp vitality failures during the 30–36-month follow-up period. Despite these limitations, this randomized controlled trial provides clinically relevant evidence regarding the use of a conventional glass ionomer liner after SCR-SD.

The primary null hypothesis was not rejected because restoration survival did not differ significantly between the treatment groups. In contrast, the secondary null hypothesis was rejected because pulp vitality preservation was significantly higher in the SE group than in the GIC + SE group. Within the limitations of this study, placement of a conventional glass ionomer liner after SCR-SD provided no detectable advantage in restoration survival and was not associated with better pulp vitality preservation over 30–36 months. Composite resin restoration using universal adhesive applied in selective enamel etching mode without a GIC liner therefore appears to be a viable restorative strategy following SCR-SD in deep caries lesions of permanent posterior teeth.

## Data Availability

No datasets were generated or analysed during the current study.
